# Dl-3-n-butylphthalide inhibits neuroinflammation by stimulating foxp3 and Ki-67 in an ischemic stroke model

**DOI:** 10.18632/aging.202338

**Published:** 2021-01-10

**Authors:** Xi Liu, Runzhe Liu, Dongxu Fu, Hao Wu, Xin Zhao, Yi Sun, Meng Wang, Xiaoping Pu

**Affiliations:** 1 National Key Research Laboratory of Natural and Biomimetic Drugs, Peking University, Beijing 100191, P.R. China; 2 Department of Molecular and Cellular Pharmacology, School of Pharmaceutical Sciences, Peking University, Beijing 100191, P.R. China; 3 CAS Key Laboratory for Biomedical Effects of Nanomaterials and Nanosafety, Institute of High Energy Physics, Chinese Academy of Sciences, Beijing 100049, P.R. China

**Keywords:** Dl-3-n-butylphthalide, ischemia, neuroinflammation, MALDI-TOF MSI, LA-ICP MSI

## Abstract

Dl-3-n-butylphthalide (NBP) has been widely used to treat ischemic stroke in China. To investigate the mechanisms underlying NBP activity, we established a permanent middle cerebral artery occlusion (pMCAO) rat model and injected the rats with 4 mg/kg/d NBP for nine days. We then assessed neuroinflammation, neovascularization and nerve regeneration within the brain. Matrix-assisted laser desorption ionization time-of-flight mass spectrometry imaging (MALDI-TOF MSI) was used to determine the phospholipid distribution, while laser ablation-inductively coupled plasma mass spectrometry imaging (LA-ICP MSI) was used to measure Foxp3, Ki-67 and pCREB levels in the brain. Immunohistochemistry was used to investigate the expression of NLR family pyrin domain containing 3 (NLRP3) and its inflammatory products, caspase-1 and interleukin-1β, in brain tissues. NBP attenuated ischemic damage and ameliorated neurological deficits in rats with pMCAO. In the ischemic brain region, NBP reduced phosphatidylethanolamine (18:0), NLRP3, caspase-1 and interleukin-1β levels, but increased levels of Foxp3, Ki-67, pCREB and several phospholipids. In molecular docking analyses, NBP bound to NLRP3, interleukin-1β, caspase-1, Foxp3, and Ki-67. These results demonstrate that NBP reduces neuroinflammation in brain tissues and promotes nerve and blood vessel regeneration, thus protecting neuromorphology and function.

## INTRODUCTION

With over two million new cases annually, stroke is associated with the most disability-adjusted life-years lost of any disease in China [1]. Ischemic stroke is mainly caused by the occlusion of cerebrovascular vessels, which diminishes the blood oxygen supply and induces the necrosis of brain tissues. Dl-3-n-butylphthalide (NBP) was first discovered in the seeds of *Apium graveolens Linn*, and was approved as an anti-ischemic stroke drug by the National Medical Products Administration in China in 2002 [2]. A number of studies have shown that NBP can improve post-stroke symptoms by ameliorating inflammation, collateral circulation, mitochondrial function, apoptosis and oxidative stress [3]. NBP alleviates experimental autoimmune encephalomyelitis by suppressing PGAM5-induced necroptosis and inflammation in microglia [4]. In addition, NBP reduces lipopolysaccharide-induced depressive-like behavior in rats through the nuclear factor erythroid 2-related factor 2 and nuclear factor κB pathways [5]. Geng et al. investigated the differentially expressed metabolites associated with NBP treatment in hippocampal tissues from a lipopolysaccharide-induced rat model of depression, and found that most of the metabolites were derived from amino acids, lipids, energy metabolism and oxidative stress [6].

Although the above studies have demonstrated the anti-inflammatory effects of NBP, few studies have used *in situ* animal stroke models to assess the mechanisms of NBP activity. In a previous study, we used matrix-assisted laser desorption ionization time-of-flight mass spectrometry imaging (MALDI-TOF MSI) to evaluate the effects of NBP on small molecules in the brains of permanent middle cerebral artery occlusion (pMCAO) model rats. We discovered that NBP prevented the abnormal accumulation of glucose and citric acid, enhanced adenosine triphosphate metabolism, improved the glutamate-glutamine cycle, increased the antioxidant content and enhanced the balance of metal ions in the brains of these rats [7].

Inflammation is an important contributor to the overall pathogenesis of ischemic stroke. Studies in animal models have revealed that innate and adaptive immune responses occur minutes to weeks or even months after ischemic stroke injury [4]. Cerebral ischemia can trigger inflammatory responses, including the activation of microglia, macrophages, neutrophils and dendritic cells [8]. The release of proinflammatory factors then induces the death of neurons and glial cells [9]. Systemic effects of stroke were observed in an MCAO-induced stroke model in mice, including greatly increased inflammatory cytokine levels during the initial activation phase, followed by severe immunosuppression linked to atrophy of the spleen and thymus [10]. Inflammatory cytokines such as interleukin (IL)-1β, E-selectin and vascular adhesion molecules are commonly measured in stroke studies, since they are known to impede recovery and correlate with an increased damage volume [11].

In this study, we further investigated the effects of NBP on inflammation and nerve and blood vessel regeneration using MALDI-TOF MSI, laser ablation-inductively coupled plasma mass spectrometry imaging (LA-ICP MSI), immunohistochemistry and molecular docking analyses. For the first time, we report the effects of NBP on the levels of phospholipids (potential biomarkers of inflammation), NLR family pyrin domain containing 3 (NLRP3), Caspase-1, IL-1β, forkhead box p3 (Foxp3), Ki-67 and phosphorylated cyclic adenosine monophosphate response element-binding protein (pCREB) in the ipsilateral hemisphere of the brain in an animal stroke model.

## RESULTS

### NBP improves functional outcomes and attenuates neurological deficits after pMCAO

**Figure 1:**
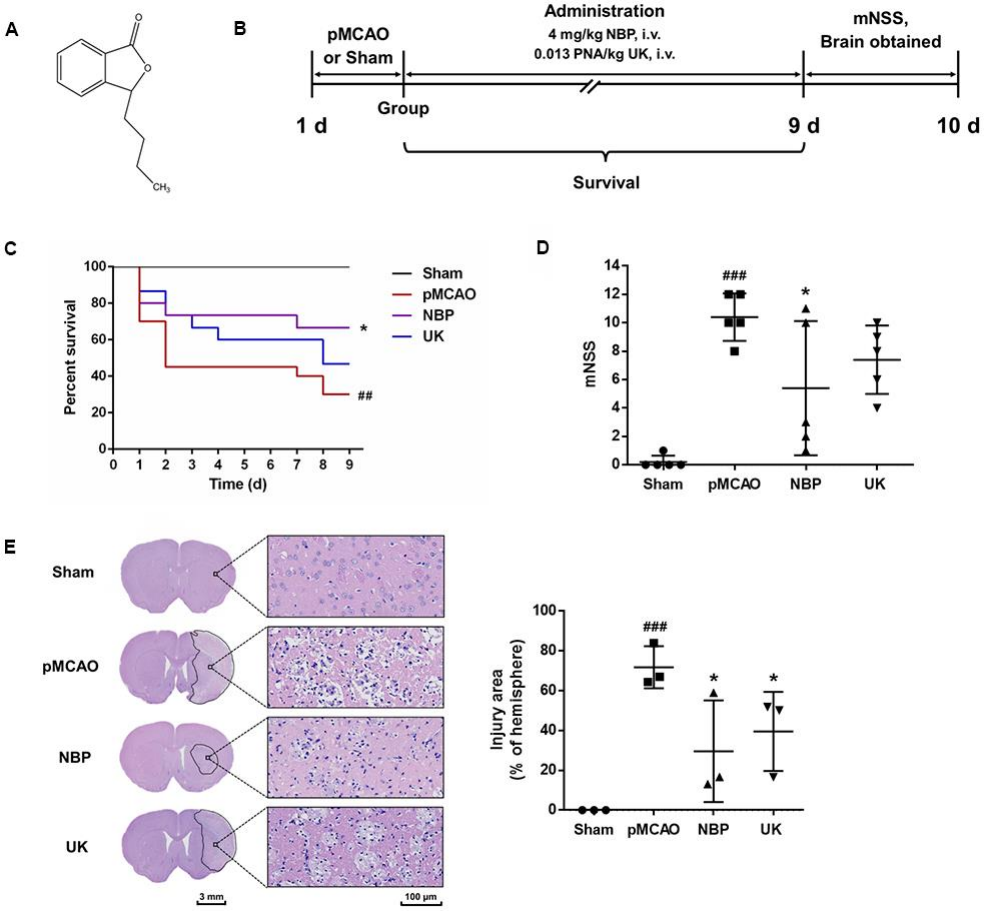
**NBP treatment attenuates brain injury after pMCAO.** (**A**) Structure of dl-NBP. Racemic NBP was used in this study. (**B**) Experimental design. The pMCAO model was established, the rats were grouped, and then the drugs were administered via the tail vein for nine days. (**C**) The survival of each group. ### *P* < 0.001 vs. the sham group, * *P* < 0.05 vs. the pMCAO group in a Log-rank (Mantel-Cox) test. (**D**) The mNSS of each group. The data are presented as the mean ± SD, n = 5, and were assessed using one-way ANOVA. (**E**) Representative images of the brain morphology and statistical analysis of the injury area following hematoxylin and eosin staining. The striatum on the lesioned side was scanned at 200× magnification, as shown on the right. Scale bar = 3 mm for the full coronal section; 100 μm for microscopic observation. The injury area was delineated using Motic DSAssistant Lite and analyzed with Image J. ### *P* < 0.001 vs. the sham group (*P* = 0.0009, pMCAO vs. Sham), * *P* < 0.05 vs. the pMCAO group (*P* =0.0161, NBP vs. pMCAO; *P* = 0.0488 UK vs. pMCAO) in one-way ANOVA, n = 3.

Racemic NBP, a natural compound extracted from celery seeds, has been approved for the clinical treatment of ischemic stroke (Figure 1A). In this study, rats were subjected to pMCAO surgery to induce stroke symptoms, and then were divided into four groups to be treated with saline (the pMCAO group), NBP, urinary kallidinogenase (UK, a positive control) for nine consecutive days (Figure 1B). An additional group was subjected to a sham surgery and treated with saline. The mortality rate was 70.0% in the pMCAO group, 33.3% in the NBP-treated group, and 53.3% in the UK-treated group (Figure 1C). The NBP group survived significantly longer than the pMCAO group (*P* < 0.05).

Ten days after surgery, the neurological function of each rat was evaluated using Longa’s method. As shown in Figure 1D, the modified neurological severity score (mNSS) was greater in the pMCAO group than in the sham surgery group (*P* < 0.001), reflecting the severe neurobehavioral damage in the pMCAO group; however, NBP significantly attenuated the neurological deficits in rats with pMCAO (*P* < 0.05). Additionally, 10 days after surgery, the striatal area of the sham surgery group exhibited a normal morphology, neat arrangement, rich cytoplasm and central nuclei. In contrast, in the pMCAO group, the striatal area of the ischemic region contained a large area of cell lysis, vacuoles and nuclear pyknosis. This region exhibited significant improvement and less cell lysis in the NBP- and UK-treated groups (Figure 1D; both *P* < 0.05 compared with the pMCAO group).

### NBP alters the levels of phospholipids in the brain

Next, we used MALDI-TOF MSI to measure various metabolites in the brain (Supplementary Figure 1). In the ischemic brain region, the pMCAO group exhibited abnormal glucose and citric acid accumulation, reduced antioxidant levels and impaired adenosine triphosphate metabolism and glutamate-glutamine cycling. NBP alleviated each of these changes, consistent with the findings of our previous study [7].

**Figure 2:**
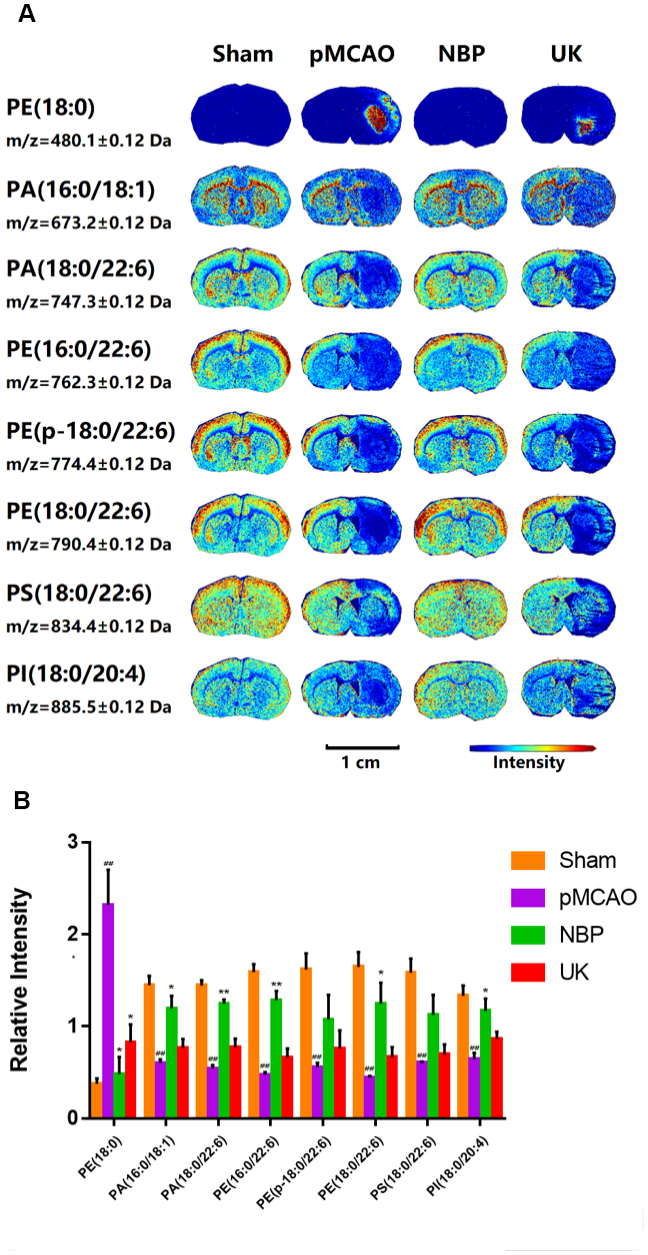
**Changes in phospholipid levels in the brains of rats with pMCAO.** (**A**) *In situ* MALDI-TOF MSI of phosphatidylethanolamine (PE) (18:0), phosphatidic acid (PA) (16:0/18:1), PA (18:0/22:6), PE (16:0/22:6), PE (p-18:0/22:6), PE (18:0/22:6), phosphatidylserine (PS) (18:0/22:6) and phosphatidylinositol (PI) (18:0/20:4). The spatial resolution was set to 100 μm. Scale bar = 1 cm. (**B**) Statistical analysis of the relative intensities of the phospholipids mentioned above. Values were normalized to those in the left brain where there was no ischemia. The data are presented as the mean ± SD, n = 3, and were assessed using one-way ANOVA. ## *P* < 0.01 vs. the sham group, * *P* < 0.05 vs. the pMCAO group, ** *P* < 0.01 vs. the pMCAO group.

Phospholipids are important components of cell membranes, and changes in their distribution can indicate the occurrence of apoptosis, necrosis and excessive inflammation in cerebral ischemia. To evaluate the effects of NBP on the brain, we used MALDI-TOF MSI to detect the distribution of phospholipid molecules in the brain. The phospholipids measured in this study included phosphatidylethanolamines, phosphatidic acids, phosphatidylserine and phosphatidylinositol. We found that NBP treatment could increase phosphatidic acid (16:0/18:1), phosphatidic acid (18:0/22:6), phosphatidylethanolamine (16:0/22:6), phosphatidylethanolamine (p-18:0/22:6), phosphatidylethanolamine (18:0/22:6), phosphatidylserine (18:0/22:6) and phosphatidylinositol (18:0/20:4) levels in the ischemic area (Figure 2A, 2B). Interestingly, only phosphatidylethanolamine (18:0) levels were elevated in the ischemia region while NBP could significantly reduced its distribution in the ischemic region after pMCAO.

### NBP inhibits the activation of the NLRP3 inflammasome

A previous study has demonstrated that NBP treatment could inhibit inflammasome activation in the Alzheimer's disease brain [12]. Upon NLRP3 inflammasome activated, pro-Caspase-1 would be transformed into Caspase-1, and then cleaved pro-IL-1β into mature IL-1β [13–15].

**Figure 3:**
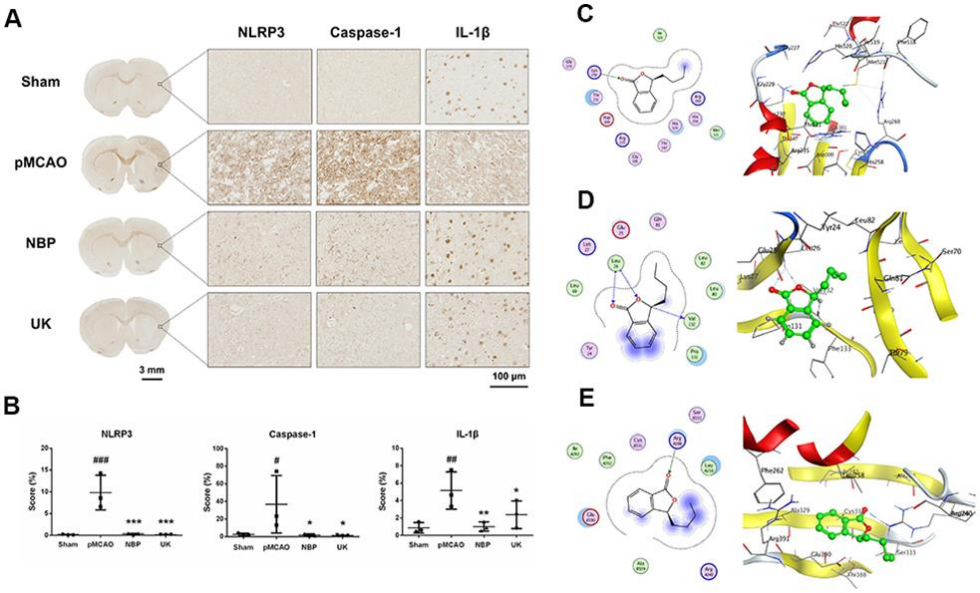
**NBP inhibits NLRP3 inflammasome activation in the right striatum of the rat brain following pMCAO.** (**A**) Representative NLRP3, Caspase-1 and IL-1β immunohistochemistry results are shown for the right striatum on the lesioned side at 200× magnification. Scale bar = 3 mm for the full coronal section; 100 μm for microscopic observation. (**B**) The score was calculated based on the percentage contribution of positive expression using IHC_Profiler in Image J. The data are presented as the mean ± SD, and were assessed using one-way ANOVA. # *P* < 0.05 vs. the sham group, ## *P* < 0.01 vs. the sham group, ### *P* < 0.001 vs. the sham group, * *P* < 0.05 vs. the pMCAO group, ** *P* < 0.01 vs. the pMCAO group, *** *P* < 0.001 vs. the pMCAO group, n = 3. Molecular docking of NBP to (**C**) NLRP3, (**D**) IL-1β, (**E**) Caspase-1.

So, we used immunohistochemistry to detect NLRP3, Caspase-1 and IL-1β levels in the right striatum of ischemia region. As predicted, NBP treatment could significantly repressed these three proteins levels in the right striatum (Figure 3A, 3B).

Next, we performed molecular docking analyses to predict whether NBP could bind to NLRP3, Caspase-1, or IL-1β. The docking score (S value) of NBP with NLRP3 protein (PDB ID: 6NPY) was -5.671, and the binding free energy of the refined docking result was -61.933 kcal/mol, indicating that (3S)-butylphthalide can bind to NLRP3 (Figure 3C and Table 1). The docking score (S value) of butylphthalide with IL-1β protein (PDB ID: 5R8M) was -5.426, and the binding free energy of the refined docking result was -33.274 kcal/mol (Figure 3D and Table 1), suggesting that (3S)-butylphthalide may bind weakly to IL-1β. The docking score (S value) of butylphthalide with Caspase-1 protein (PDB ID: 2FQQ) was -4.232, and the binding free energy of the refined docking result was -43.720 kcal/mol, indicating that (3S)-butylphthalide can bind to Caspase-1 (Figure 3E and Table 1).

**Table 1:** List of the docking research.

**ID**	**Molecules**	**Source**	**Target**	**PDB ID**	**Docking score**	**Binding free energy(kcal/mol)**
1	(3S)-butylphthalide	Homo	NLRP3	6NPY	-5.671	-61.933
2	(3S)-butylphthalide	Homo	IL-1B	5R8M	-5.426	-33.274
3	(3S)-butylphthalide	Homo	Caspase-1	2FQQ	-4.232	-43.720
4	(3S)-butylphthalide	Homo	Foxp3	4WK8	-5.0941	-44.321
5	(3S)-butylphthalide	Homo	Ki-67	2AFF	-5.256	-41.565

### NBP increases Foxp3, Ki-67 and pCREB levels after cerebral ischemia

**Figure 4:**
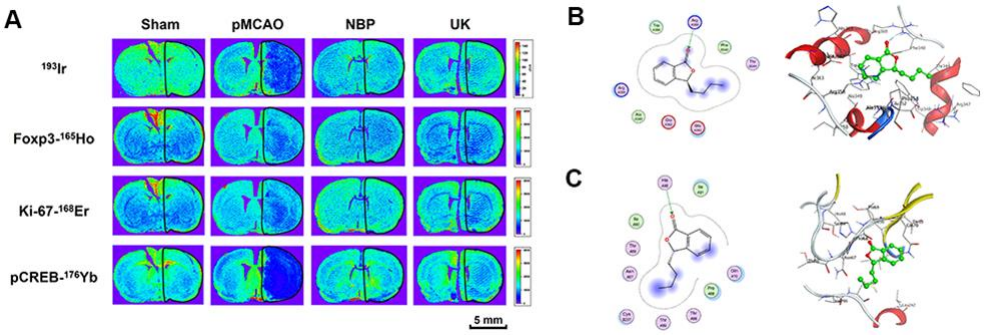
**NBP treatment improved Foxp3, Ki-67 and pCREB levels in the ischemia region with pMCAO.** (**A**) For all tissues, ^193^Ir and the three metal-labeled proteins were measured simultaneously at a resolution of 110 μm. Scale bars = 5 mm. Molecular docking of to (**B**) Foxp3, (**C**) Ki-67.

In addition, we used LA-ICP MSI to measure Foxp3, Ki-67 and pCREB levels in the right cortex and striatum. NBP treatment could increase these proteins levels in the cortical and striatal regions of the ischemic brain (Figure 4A). NBP treatment could also reduce cell death in the ischemia region, as further demonstrated by ^193^Ir, an intercalator dye that specifically binds to the nucleus.

Further, we also performed docking molecular analysis between NBP and the two target Foxp3 and Ki-67. Since foxp3 and Ki-67 don’t have complete crystal structures, we used partial sequences of the two target for docking analysis. The total length of Foxp3 is 431 amino acids, of which only the 336-417 part of the sequence has the structure resolved. We used this part of the structure for docking analysis. The docking score (S value) of butylphthalide with protein Foxp3 (PDB ID: 4WK8) is -5.0941 and the binding free energy of refined docking result is -44.321 kcal/mol. This computational result indicated that (3S)-butylphthalide may have a very weak interaction with Foxp3 (Figure 4B and Table 1). As for Ki-67, it is consisted with 3256 amino acids, of which the 1-120 and 496-536 sequences have crystal structures, so we used a longer sequence structure of 1-120 for docking analysis. The docking score (S value) of butylphthalide with protein ki-67 (PDB ID: 2AFF) is -5.256, and the binding free energy of refined docking result is -41.565 kcal/mol, indicating that (3S)-butylphthalide may have a weak interaction with ki-67 (Figure 4C and Table 1).

## DISCUSSION

MCAO is a common model of ischemic stroke. The modified Longa method was used to prepare the pMCAO model in the present study, as this method is simple, enables stable and reproducible modeling, and provides a good simulation of clinical stroke caused by ischemia [16]. The mNSS test is the preferred method of assessing the extent of neurological impairment in permanent and transient MCAO models. In our experiments, pMCAO rats displayed obvious symptoms of neurological damage, while NBP improved the degree of neurological damage in pMCAO rats to some extent. Together with the pathological staining results in brain tissues, these data demonstrated that NBP significantly reduced the area of brain damage area in the pMCAO model, consistent with previously published results [17].

Brain damage in ischemia mainly results from the consumption of oxygen and energy (within a few minutes), the release of excitatory amino acids (within a few hours), the inflammatory response and apoptosis. As these biochemical reactions induce the death of nerve cells in brain tissue, it is important to prevent them during the early treatment of cerebral infarction. To study the effects of NBP on cerebral ischemia, we employed two MSI techniques to observe molecules of interest in brain slices, namely MALDI-TOF MSI and LA-ICP MSI. LA-ICP MSI is generally used for the *in situ* analysis of trace elements, and can be used for simultaneous protein imaging in combination with immunohistochemistry [18, 19]. In this method, several metal-labeled antibodies are applied to bind to specific proteins on a tissue slice, and then the tissue is ablated by a laser to produce an aerosol, which is transported by a carrier gas into plasma to complete ionization. Then, the ions are detected by mass spectrometry, and a distribution map of various proteins in a single test is obtained. LA-ICP MSI can provide highly multiplexed imaging of proteins in tissue sections at the single-cell level, and is gradually becoming a widely used imaging method in biomedical research [20].

After cerebral ischemia, a severe inflammatory reaction occurs in the brain tissue. In addition to inducing cell pyroptosis, excessive inflammatory factors promote apoptosis and necrosis [21]. In neurons and glial cells, the NLRP3 inflammasome may be important for detecting cell damage and inducing inflammatory responses [22, 23]. During the innate immune response, the NLRP3 inflammasome activates Caspase-1, which then promotes the maturation of the cytokine pro-IL-1β. Additionally, the NLRP3 inflammasome induces Caspase-1-dependent pyroptosis and cell death under pathological conditions of inflammation and stress [24]. Using immunohistochemistry, we demonstrated that NBP reduced the levels of NLRP3, IL-1β and Caspase-1 in the ischemic brain area, indicating that NBP can inhibit the inflammatory response induced by NLRP3. Similarly, Yang et al. reported that NBP ameliorated neurovascular inflammation and ischemic brain injury in mice. NBP reduced the infiltration of myeloid cells into the brain and improved cerebral blood flow after reperfusion [25].

Using LA-ICP MSI, we also found that NBP increased the distribution of Ki-67 and pCREB in the ischemic cortex and striatum. The most well-recognized mechanisms of NBP activity are improving the microcirculation, promoting angiogenesis and increasing cerebral blood flow in the ischemic area. Ki-67 is a nuclear protein involved in cell proliferation, and has been used as a marker of vessel density [26]. We found that NBP treatment increased Ki-67 levels in the ischemic area in our pMCAO model, illustrating that NBP can improve angiogenesis in the ischemic region. NBP has also been reported to promote the expression of vascular endothelial growth factor and angiopoietin-1, thus inducing angiogenesis [27].

T_reg_ cells are a special subset of T cells that induce and maintain the stability and tolerance of the immune environment, thus preventing the overactivation of the immune system in inflammatory diseases [28–30]. The development and function of T_reg_ cells depend on the transcription factor Foxp3; thus, Foxp3 deficiency can suppress T_reg_ cell function [28, 31, 32]. Foxp3(+) T cells are typical anti-inflammatory cells that govern antigen-specific immune responses and immune tolerance [33, 34]. A previous report described staining for Foxp3 in the striatal and cortical regions of the brain after ischemia and reperfusion in an MCAO-induced stroke model [35]. In our study, Foxp3 levels were detected using LA-ICP MSI, which can measure the expression of several different proteins in a single test. We found for the first time that NBP significantly increased Foxp3 expression in the ischemic area in an animal stroke model. NBP may exert anti-inflammatory and neuroprotective effects by maintaining the function of Foxp3(+) T cells.

CREB is a transcription factor that promotes synaptic plasticity, memory and cognition [36]. The activation of the CREB pathway induces nerve regeneration after cerebral ischemia-reperfusion in rats [37]. Liu et al. found that silencing Gadd45b significantly reduced both brain-derived neurotrophic factor and cyclic adenosine monophosphate/protein kinase A/pCREB levels and promoted Rho-associated coiled-coil kinase expression, suggesting that Gadd45b stimulates recovery after stroke by enhancing axonal plasticity [38]. In the present study, LA-ICP MSI revealed that NBP increased pCREB levels in the ischemic cortex and striatum. This finding indicated that NBP can improve nerve regeneration after pMCAO, consistent with the results of Yang et al. [39]. Further research is needed to determine how NBP promotes nerve regeneration and protects cognitive function.

**Figure 5:**
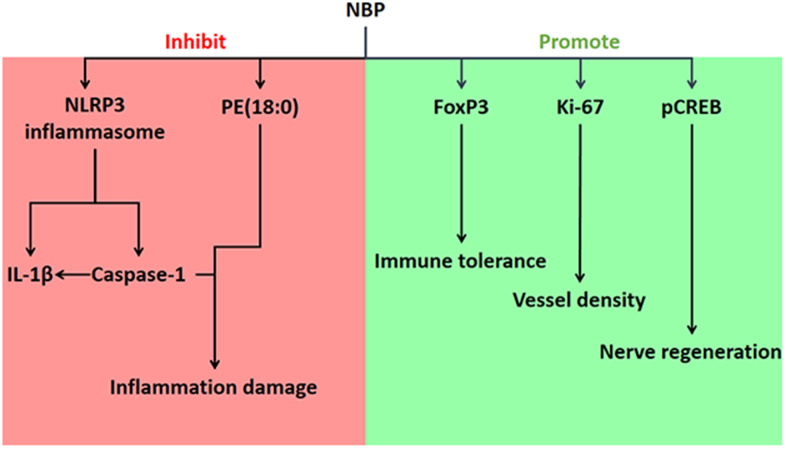
**Effects of NBP on ischemic stroke in pMCAO rats, and the associated mechanisms.**

In conclusion, we demonstrated that NBP can reduce inflammatory damage, maintain immune tolerance, improve vascular density and promote nerve cell regeneration (Figure 5). NBP improved neurobehavior, reduced the area of brain damage, alleviated inflammation and stimulated nerve regeneration and angiogenesis in a rat model of pMCAO-induced cerebral ischemia. In addition, NBP reversed the changes in various phospholipid molecules, Foxp3, Ki-67 and pCREB in the brain tissues of pMCAO rats. By inhibiting the induction of the NLRP3 inflammasome, Caspase-1 and IL-1β, NBP further suppressed inflammation and ameliorated brain tissue damage. Further research is needed to determine how NBP promotes nerve regeneration and angiogenesis.

## MATERIALS AND METHODS

### Materials

Healthy 8- to 10-week-old male Sprague Dawley rats weighing 280-310 g were purchased from Beijing Vital River Experimental Animal Technology Co., Ltd. (license number: SCXK [Beijing] 2012-0001). The rats were housed in a specific-pathogen-free laboratory at the Experimental Animal Department of Peking University Health Science Center. The rats were kept under standard environmental conditions (23 ± 1° C, humidity 45 ± 5%, 12-h light/dark cycle) and had free access to standard food (Keaoxieli, Beijing, China) and drinking water.

Injectable NBP (Figure 1A) was provided by China Shijiazhuang Pharmaceutical Company, Ltd. (batch number: 17051026). Injectable urinary kallidinogenase was purchased from Guangdong Tianpu Biochemical Pharmaceutical Co., Ltd. (batch number: 311701021).

### Model establishment and drug treatment

The pMCAO rat model was established in accordance with our previous study [7]. Rats were anesthetized with an intraperitoneal injection of 0.35 g/kg chloral hydrate. The right common carotid artery and the external carotid artery were exposed after an incision was made in the neck. Then, the right external carotid artery and the proximal end of the common carotid artery were ligated with a suture. A V-shaped cut was made at the distal end of the common carotid artery, and a thread was inserted through the incision to the internal carotid artery. When the thread had been inserted 15-16 mm into the internal carotid artery and the resistance increased significantly, the insertion was stopped and the thread was tied to the distal end of the common carotid artery with a suture to fix it. The incision in the neck was then sutured and disinfected with medical-grade alcohol. The surgery was completed within 15 min, and the rats awakened after approximately 2 h. The Longa score was determined after the animals were awake, and rats with scores ≥ 2 were considered successful models [16, 40]. The sham surgery group underwent a similar operation, but the common carotid artery was left intact and a thread was not inserted (n = 6).

After the pMCAO surgery, approximately 60% of the rats developed stroke symptoms and were considered successful models. These animals were randomly divided into four groups: the pMCAO group (n = 20), the NBP-treated group (n = 15), and the UK-treated group (n = 15). The latter two groups were included as positive controls. Drug administration was started 2 h after the end of modeling. In the NBP group, 4 mg/kg NBP was administered daily through the tail vein [7]; and in the UK group, 0.013 PNA/kg UK was administered daily through the tail vein. The sham surgery group and the pMCAO group were given the same volume of normal saline daily. The drugs were administered for nine days. At the end of drug administration, six rats were randomly selected from each group for the following studies (Table 2).

**Table 2:** The number of rats in each group.

**Total rats in each group**	**Day 1**	**Day 10**
Sham	6	6
pMCAO	20	6
NBP	15	10
UK	15	7

### Behavioral test

The mNSS test was used to evaluate the degree of neurological impairment after the establishment of the rat pMCAO model. The evaluation included exercise, sensory, reflex and balance tests. The mNSS score was determined according to the method of Chen (Supplementary Table 1) [41]. A higher mNSS score indicated more severe neurobehavioral damage.

### MALDI-TOF MSI for the detection of small molecules in brain tissues

Three rats from each group were perfused with normal saline, and their brains were quickly collected, frozen in liquid nitrogen and stored at -80° C. The frozen brain tissues were sliced into 10-μm-thick sections beginning 0.6 mm from the bregma, and were then mounted on the indium tin oxide-coated surface of a glass slide for MALDI-TOF MSI. MALDI-TOF MSI was conducted and analyzed according to the method described by Liu [42]. An ultrafleXtreme MALDI-TOF/TOF mass spectrometer (Bruker Daltonics, Billerica, MA, USA) equipped with a Smartbeam Nd: YAG 355 nm laser was used for MALDI analysis. Negative-ion mass spectra were acquired in reflector mode with a pulsed ion extraction time of 80 ns, an accelerating voltage of 20.00 kV, an extraction voltage of 17.90 kV, a lens voltage of 5.85 kV and a reflector voltage of 21.15 kV. For MSI analysis, the imaging spatial resolution of the rat brain tissues was set to 100 μm. The regions of interest were manually defined in the imaging software using both the optical image and the MSI data image.

### Histopathological observation

The other three rats from each group were perfused with normal saline and formalin, and their brains were fixed in formalin overnight. Then, 4-μm-thick paraffin slices 0.6 mm from the bregma were prepared for hematoxylin and eosin staining. Pathological changes in the brain were observed using a Motic Tele-Microscope System (Motic China Group, Xiamen, China).

### Immunohistochemistry

For immunohistochemistry, formalin-fixed, paraffin-embedded brain tissue sections were stained with prediluted antibodies, including anti-NLRP3 (1:100 dilution), anti-Caspase-1 (1:50 dilution) and anti-IL-1β (1:100 dilution), according to standard protocols [43]. Briefly, the sections were baked, dewaxed and rehydrated as described above. Heat-induced epitope retrieval was conducted in sodium citrate (pH 6) in a 96° C water bath for 30 min. After immediate cooling, the sections were washed with deionized water and PBS for 5 min each. The slides were incubated with 3% H_2_O_2_ in PBS for 1 h at 37° C and then washed three times for 5 min with PBS. The sections were blocked with 10% normal serum/0.3% Triton X-100 in PBS for 1 h at 37° C, and then were incubated overnight at 4° C with the primary antibody (diluted in PBS/1% bovine serum albumin). The samples were then washed three times for 5 min with PBS. The sections were incubated with horseradish peroxidase-conjugated goat anti-rabbit IgG H&L as the secondary antibody for 1 h at 37° C. After three additional 5-min washes with PBS, immunoperoxidase staining was developed using a 3,3’-diaminobenzidine chromogen (Dako) for 5 min. The slides were washed with deionized water for 5 min, dehydrated in graded alcohol and xylene, mounted and coverslipped. Changes in the brain were observed under an optical microscope (Motic Tele-Microscope System).

### LA-ICP MSI to observe the distribution of Foxp3, Ki-67 and pCREB in brain tissues

Staining for Foxp3, Ki-67 and pCREB was performed manually (antibody list in Table 3). Formalin-fixed, paraffin-embedded brain tissues were sectioned at a thickness of 4 μm for LA-ICP MSI [44]. The tissue sections were baked for 2 h at 60° C in a slide oven. Then, the tissue sections were dewaxed in fresh xylene for 20 min and rehydrated in a graded series of alcohols (absolute ethanol, and 95:5, 80:20, 70:30 and 0:100 ethanol:deionized water; 5 min each). Heat-induced epitope retrieval was conducted in Tris-ethylenediaminetetraacetic acid buffer (pH 9) in a 96° C water bath for 30 min. After immediate cooling, the sections were washed with deionized water and phosphate-buffered saline (PBS) for 10 min each and then blocked with 3% bovine serum albumin in PBS for 45 min. The sections were incubated overnight at 4° C with an antibody master mix containing a 1:50 dilution of anti-Foxp3 (FJK-16s)-165Ho, a 3:100 dilution of anti-Ki-67 (B56)-168Er and a 1:100 dilution of anti-pCREB [S133] (87G3)-176Yb. After two 8-min washes in 0.2% Triton X-100 in PBS and two 8-min washes in PBS, the sections were incubated with Cell-ID^TM^ Intercalator-Ir (125 μM) in PBS (1:400 dilution) for 30 min at room temperature. After being washed, the sections were dried at room temperature before LA-ICP MSI.

**Table 3:** Antibodies used in this study.

**Antibody**	**Source**	**Identifier**
Cell-IDTM Intercalator-Ir	Fluidigm	Cat# 201192A
Anti-Mouse/Rat Foxp3 (FJK-16s)-165Ho	Fluidigm	Cat# 3165024A
Anti-Ki-67 (B56)-168Er	Fluidigm	Cat# 3168022D
Anti-pCREB [S133] (87G3)-176Yb	Fluidigm	Cat# 3176005A
Anti-NLRP3	Abcam	Cat# ab214185
Anti-IL-1β	Abcam	Cat# ab9722
Anti-Caspase-1	Immunoway	Cat# YT5743

An NWR 213 laser ablation system (Elemental Scientific Lasers, Bozeman, MT, USA) coupled to a NexION 300D ICP-MS (Perkin Elmer, Waltham, MA, USA) was used for LA-ICP MSI analysis. Helium was used as the ablation gas, and was introduced with argon gas through a T-piece into the ICP-MS after the ablation cell. The LA-ICP-MS was calibrated with NIST 612 glass standards for high U signal intensity, while oxide production (i.e., the UO+/U+ ratio) was minimized. The LA-ICP-MS operating parameters are shown in Table 4. The laser ablation parameters (spot size, laser energy, scan rate and ablation frequency) were carefully chosen to guarantee quantitative ablation of the brain sections and minimal ablation of the glass slides. All brain sections were ablated in line ablation mode, where the ICP-MS was triggered with a laser shot. Data were acquired on the ICP-MS in time-resolved analysis mode. The acquired data were processed into images using Iolite software (V3.6, serial number: 74272) [45].

**Table 4:** Operating parameters of the LA-ICP-MS for elemental imaging.

**ICP-MS**		**Laser ablation**	
Nebulizer gas	0.94 L min-1	He carrier gas	0.60 L min-1
Auxiliary gas	0.50 L min-1	Ablation frequency	20 Hz
Plasma gas	18 .0 L min-1	Spot size	100 μm
RF power	1300 W	Scan speed	100 μm s-1
Acquisition mode	Time-resolved analysis	Fluence	2.05 J cm-2
Isotope monitored	165Ho, 168Er, 176Yb, 193Ir		
Dwell time	50 ms per isotope		

### Molecular docking

Molecular docking analyses were conducted using MOE v2019.1. The 3D structures of (3S)-butylphthalide were downloaded from the PubChem database. The 3D structures of NLRP3, IL-1β, Caspase-1, Ki-67 and Foxp3 were downloaded from the Research Collaboratory for Structural Bioinformatics Protein Data Bank. Prior to docking, the force field of AMBER10:EHT and the implicit solvation model of the Reaction Field were selected. MOE-Dock was used for molecular docking simulations of the small molecules with the targets.

Since most receptors are not part of a complex, we carefully investigated research papers describing the structures of the target proteins. We also used the MOE Site Finder to calculate possible active sites in the receptors based on the 3D atomic coordinates of the protein crystals. The Site Finder methodology is based on Alpha Shapes, which are generalizations of the convex hulls developed by Edelsbrunner in 1995. Such calculations are useful for site-directed simulation experiments to determine potential sites for ligand-binding docking calculations, for restriction set calculations to render partial molecular surfaces, and for MultiFragment Searches.

The docking workflow followed the “induced fit” protocol, in which the side chains of the receptor pocket are allowed to move according to the ligand conformation, with constraints on their positions. The weight used to tether side chain atoms to their original positions was 10. For each ligand, all docked poses were first ranked based on London dG scoring. Then, a force field refinement was carried out on the top 20 poses, and rescoring was performed using the GBVI/WSA dG scoring function. Molecular graphics were generated in MOE.

### Statistical analysis

Statistical analyses were performed using GraphPad Prism software. The data are expressed as the mean ± standard deviation (SD). The distribution of the data was assessed with the Shapiro-Wilk normality test. One-way analysis of variance (ANOVA) was used to compare the groups, and *P* < 0.05 was considered statistically significant.

## Supplementary Material

Supplementary Figure 1

Supplementary Table 1
