# Supplementary material for: Dl-3-n-butylphthalide inhibits neuroinflammation by stimulating foxp3 and Ki-67 in an ischemic stroke model
Source: Aging (Albany NY). 2021 Jan 10;13(3):3763–78. doi: 10.18632/aging.202338 (PMC7906154; doi:10.18632/aging.202338)
Supplement: Supplementary Figure 1 [file aging-13-202338-s001.pdf]

SUPPLEMENTARY FIGURE

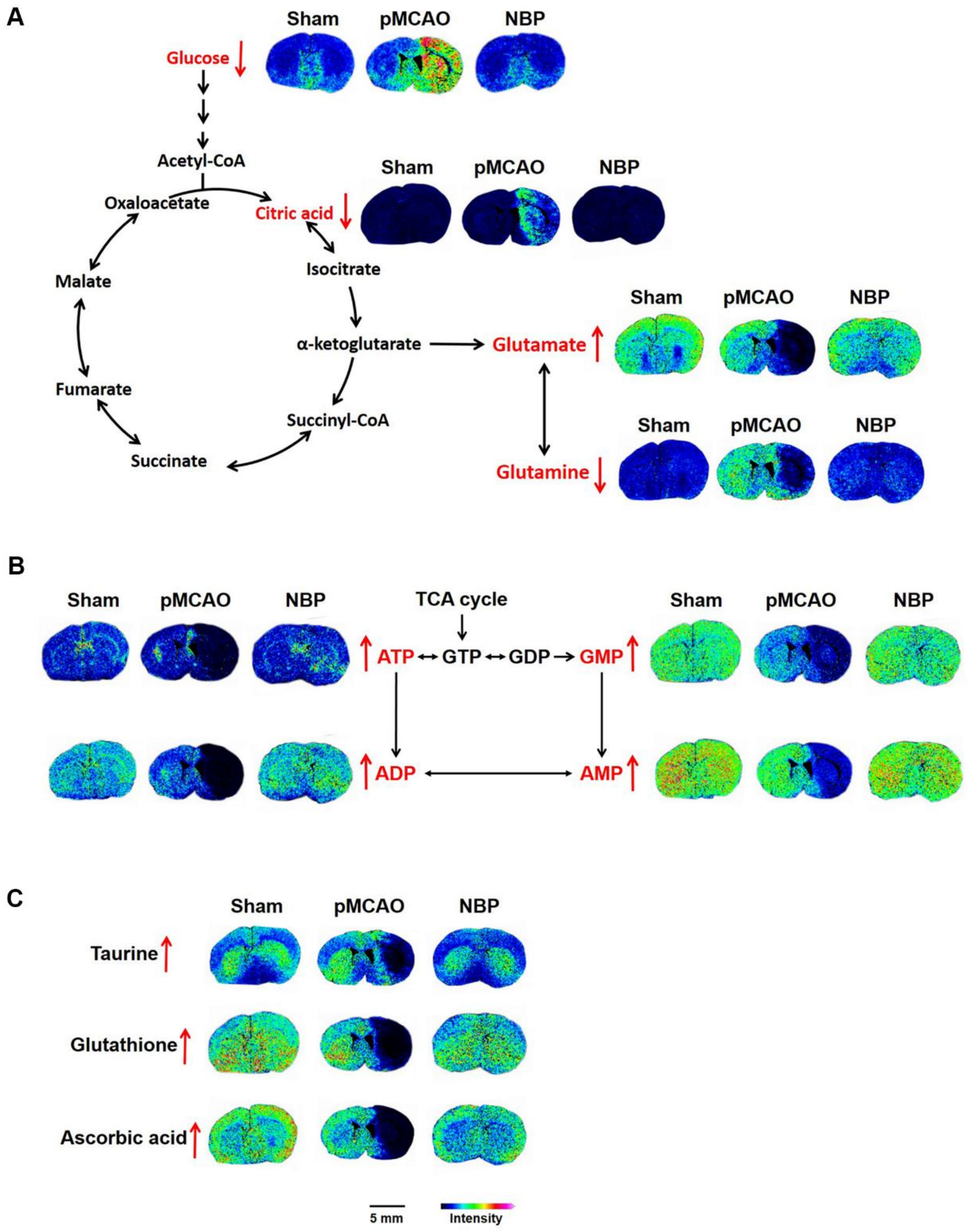

Supplementary Figure 1. NBP alleviated the abnormal accumulation of glucose and citric acid, improved the levels of metabolites involved in the glutamate-glutamine cycle. (**A**), enhanced ATP metabolism (**B**), and increased the levels of antioxidants (**C**) in a rat model of pMCAO. MALDI-TOF-MS imaging of glucose (215 ± 0.2 Da), citric acid (191.05 ± 0.1 Da), glutamate (146.07 ± 0.1 Da), glutamine (145.07 ± 0.1 Da), ATP (505.85 ± 0.1 Da), ADP (425.97 ± 0.1 Da), AMP (346.01 ± 0.1 Da), GMP (362.01 ± 0.1 Da), taurine (124.05 ± 0.1 Da), glutathione (306.05 ± 0.1 Da), and ascorbic acid (175.05 ± 0.1 Da). The spatial resolution was set to 100 μm. Scale bar = 5 mm. Sham: sham surgery group; pMCAO: permanent middle cerebral artery occlusion group; NBP: dl-3-n-butylphthalide-treated group.
