# Supplementary material for: Dl-3-n-butylphthalide inhibits neuroinflammation by stimulating foxp3 and Ki-67 in an ischemic stroke model
Source: Aging (Albany NY). 2021 Jan 10;13(3):3763–78. doi: 10.18632/aging.202338 (PMC7906154; doi:10.18632/aging.202338)
Supplement: Supplementary Table 1 [file aging-13-202338-s002.pdf]

SUPPLEMENTARY TABLE

Supplementary Table 1. Modified neurological severity score points.

Motor tests	
Raising rat by tail	3
Flexion of forelimb	1
Flexion of hindlimb	1
Head moved 10° to vertical axis within 30 s	1
Placing rat on floor (normal 0; maximum 3)	3
Normal walk	0
Inability to walk straight	1
Circling toward paretic side	2
Falls down to paretic side	3
Sensory tests	2
Placing test (visual and tactile test)	1
Proprioceptive test (deep sensation, pushing paw against table edge to stimulate limb muscles)	1
Beam balance tests (normal 0; maximum 6)	6
Balances with steady posture	0
Grasps side of beam	1
Hugs beam and 1 limb falls down from beam	2
Hugs beam and 2 limbs fall down from beam, or spins on beam (60 s)	3
Attempts to balance on beam but falls off (40 s)	4
Attempts to balance on beam but falls off (20 s)	5
Falls off; no attempt to balance or hang on to beam (20 s)	6
Reflex absence and abnormal movements	4
Pinna reflex (head shake when auditory meatus is touched)	1
Corneal reflex (eye blink when cornea is lightly touched with cotton)	1
Startle reflex (motor response to a brief noise from snapping a clipboard paper)	1
Seizures, myoclonus, myodystony	1
Maximum points	18

One point is awarded for inability to perform the tasks or for lack of a tested reflex: 13–18, severe injury; 7–12, moderate injury; 1– 6, mild injury.
